# MHC-dependent mate choice is linked to a trace-amine-associated receptor gene in a mammal

**DOI:** 10.1038/srep38490

**Published:** 2016-12-12

**Authors:** Pablo S. C. Santos, Alexandre Courtiol, Andrew J. Heidel, Oliver P. Höner, Ilja Heckmann, Martina Nagy, Frieder Mayer, Matthias Platzer, Christian C. Voigt, Simone Sommer

**Affiliations:** 1 Leibniz Institute for Zoo and Wildlife Research (IZW) Berlin, Germany; 2 Institute of Evolutionary Ecology and Conservation Genomics, University of Ulm, Ulm, Germany; 3 Berlin Center for Genomics in Biodiversity Research (BeGenDiv), 14195 Berlin, Germany; 4 Leibniz Institute on Age - Fritz Lipmann Institute, Jena, Germany; 5 Museum für Naturkunde, Leibniz Institute for Research on Evolution and Biodiversity, Berlin, Germany

## Abstract

Major histocompatibility complex (MHC) genes play a pivotal role in vertebrate self/nonself recognition, parasite resistance and life history decisions. In evolutionary terms, the MHC’s exceptional diversity is likely maintained by sexual and pathogen-driven selection. Even though MHC-dependent mating preferences have been confirmed for many species, the sensory and genetic mechanisms underlying mate recognition remain cryptic. Since olfaction is crucial for social communication in vertebrates, variation in chemosensory receptor genes could explain MHC-dependent mating patterns. Here, we investigated whether female mate choice is based on MHC alleles and linked to variation in chemosensory trace amine-associated receptors (TAARs) in the greater sac-winged bat (*Saccopteryx bilineata*). We sequenced several MHC and TAAR genes and related their variation to mating and paternity data. We found strong evidence for MHC class I-dependent female choice for genetically diverse and dissimilar males. We also detected a significant interaction between mate choice and the female TAAR3 genotype, with TAAR3-heterozygous females being more likely to choose MHC-diverse males. These results suggest that TAARs and olfactory cues may be key mediators in mammalian MHC-dependent mate choice. Our study may help identify the ligands involved in the chemical communication between potential mates.

The ability to discriminate among potential mating partners has been selected for in a wide range of organisms, as the fitness benefits of choosing a particular mate often exceed the costs associated with mate choice^1^. Because females usually are responsible for offspring care and have a lower potential reproductive rate than males, they tend to be the choosy sex^2^,^3^. Females are known to choose males based on traits indicating their quality such as the amount and quality of resources (territory, food, care) provided by them and their health status^3^. Over the past decades, evidence has accumulated that females of many species consider a male’s genetic constitution as an additional indicator of his quality and choose males according to alleles from specific loci of the major histocompatibility complex (MHC)^4^,^5^. Little is known, however, about the mechanisms that females use to assess male alleles and compare them to their own genetic repertoire.

The MHC is a highly polymorphic and gene-dense region in vertebrate genomes which harbors loci that play a central role in life history decisions, self/nonself recognition and in immune response^6^,^7^. MHC molecules are membrane receptors that, according to the current paradigm, present peptides of intracellular (MHC class I) or extracellular (MHC class II) origin to immune-surveillance-dedicated cells such as T lymphocytes^6^. As a consequence, an individual’s ability to recognize and resist a broad spectrum of potential pathogens tends to correlate with the diversity of its MHC alleles^8^,^9^ (but see the study by Milinski^10^). In this context, the evolutionary benefits of MHC-dependent mate choice are straightforward: choosy individuals preferring MHC-dissimilar mates are expected to produce offspring with higher fitness. In fact, various studies found that MHC-dependent mate choice had a positive effect on parasite load and survival rates of juveniles^11^,^12^ and also ultimately on offspring fitness^13^. Except for some recently published cases of MHC-assortative choice^14^,^15^, MHC-disassortative mate choice has been found in many vertebrate species, including fish^11^,^16^, reptiles^17^, birds^18^ and mammals^4^,^9^,^19^.

Olfactory signals are probably essential for communication between males and females in the context of MHC-dependent mate choice^4^,^18^,^20^,^21^. Trace amine-associated receptors (TAARs) are a class of chemosensory molecules expressed in the main olfactory epithelium^22^,^23^,^24^. They have recently gained much attention due to their role in chemical communication^24^, as receptors for psychoactive^25^ and leucocyte activating substances^26^. They are structurally similar to canonical olfactory receptors^27^ and are highly sensitive to low concentration volatile amines^27^,^28^. Many of the currently known ligands for TAARs are protein derivatives^29^, including potential pheromones observed in the urine of male rodents^24^. Polymorphisms that cause conformational differences in TAARs result in different ligand-binding profiles, which are expected to correspond to individual variation in a population^29^. Thus, a growing body of evidence suggests that the TAAR olfactory subsystem is involved in individual recognition^24^,^29^. One possible mechanism may be that TAARs bind urine-borne byproducts of polymorphic MHC molecules. If so, females with a larger TAAR gene repertoire should have an increased ability to discriminate between males based on odor cues. Despite all recent suggestive findings, we are unaware of any reports to date testing the possible role of TAARs in MHC-dependent odor recognition or mate choice behavior.

Here, we investigated MHC-dependent mate choice in a free-ranging population of the greater sac-winged bat (*Saccopteryx bilineata*, Emballonuridae) by combining detailed genetic profiling with large-scale parentage analyses of individually banded bats. This species has been demonstrated to rely on odor cues for social communication and courtship^30^,^31^. Males have a pouch-like organ on their wing membranes which they fill with an odoriferous substance made out of distinct secretions including saliva and urine^30^. Male *S. bilineata* spend up to one hour per day refilling and cleaning their wing sac liquid^32^, which is then used to attract female attention, as males fan their scents towards females while hovering in front of them. Although not all perfume components have been identified, the most abundant male-specific substances include information on species and individual identity^31^. The wing sac scent changes with the onset of sexual maturation and is likely to enclose male-specific information^33^ related to health status and MHC genotype.

Female *S. bilineata* are larger and physically superior over males and may easily visit any prospective male mating partner in a colony^34^. Additionally, males are incapable of monopolizing access to females during nocturnal foraging, making it likely that females choose freely among available mates^35^. Earlier investigations on the roosting ecology of *S. bilineata* revealed that colonies consist of one or several basic social units each consisting of a single adult male and up to eight females, so-called harems^36^. The observation that approximately 70% of offspring are sired by other harem and non-harem males rather than the females’ own harem males further suggests that female choice takes place in this mating system^37^.

To test whether female *S. bilineata* exercise MHC-dependent mate choice, we genotyped MHC class I and class II genes using large-scale next-generation amplicon sequencing. Chiropterans are natural reservoirs of several viral diseases and host many zoonotic viruses^38^, suggesting that exposure to and prevalence of viruses is particularly high among bats^39^,^40^,^41^. Because viruses mainly elicit a response of MHC class I receptors^6^ (but mind exceptions^42^), high viral exposure and prevalence is expected to direct selection pressure more towards MHC class I than class II receptors. Thus, if female *S. bilineata* exercised MHC-dependent mate choice, we would expect a stronger statistical signal for MHC class I than class II. We additionally genotyped three TAAR loci in order to test if they influenced mate choice. Our dataset consisted of 972 successfully genotyped bats sampled over the course of sixteen consecutive years.

## Results

### Initial evaluation of genetic data

Amplicons from 972 individuals (99.1% of sampled bats) yielded reads which passed all quality and frequency filters and were assigned to alleles of at least one of the investigated genes. We found the following number of alleles for each gene: (i) MHC Class I, with 50 alleles among 447 individuals and 3–6 alleles per individual, indicating a minimum of 3 amplified loci; (ii) MHC Class II, with 25 alleles among 615 individuals and 2–4 alleles per individual, indicating a minimum of 2 amplified loci; (iii) TAAR2, with 9 alleles among 964 individuals and 1–2 alleles per individual, indicating at least 1 locus; (iv) TAAR3, with 5 alleles among 876 individuals and 1–2 alleles per individual, indicating at least 1 locus and (v) TAAR8, with 8 alleles among 884 individuals and 1–2 alleles per individual, indicating at least 1 locus. A robust assignment of alleles to individual loci was not possible by analyzing sequence phylogeny and also due to the lack of a reference sequence for our study species. Thus, we considered all putative functional sequences (null alleles and pseudogenes were filtered out by the BLAST filter) from each gene as alleles from single loci. Repeatability of allele calling was 88.2% on average (ranging from 75% for MHC-I to 100% for TAAR8, more detail in the supplements). All alleles not found in replicates were rare variants (less than 5 individuals or 0.5% of the sample). We then carried out a Mendelian segregation analysis, using family trio information, which led to the correction of the genotypes of six individuals for the MHC-I (one allele removed per individual) and two individuals for MHC-II (one allele removed per individual). No Mendelian errors were observed for any of the TAAR loci. Linkage disequilibrium (LD) was observed among the three TAAR loci (p-values < 10^−5^ for all three combinations) but not between MHC class I and II (p-value = 0.56) or between MHC and TAAR loci (p-values > 0.59 for all six combinations).

### Test of MHC-dependent female mate choice with GLMMs

**Figure 1:**
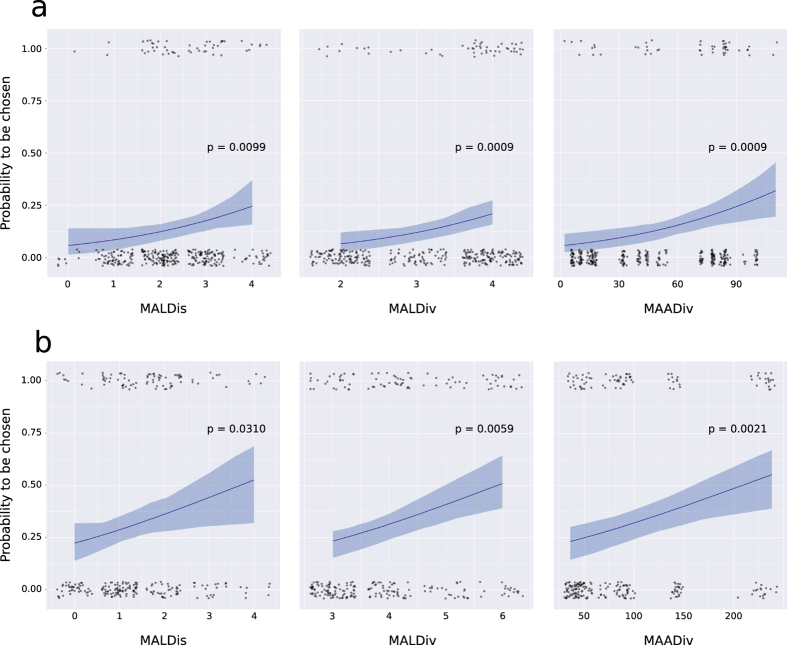
MHC-dependent female choice tested with linear modelling. Regression curves fitted by GLMMs on the probability of males to be chosen by females based on three MHC genetic parameters, MALDis, MALDiv and MAADiv. The 95% confidence intervals (shaded areas) were generated by 10^5^ bootstraps and the p-values are given above each curve. (**a**) MHC-I, 50 real couples were compared with 351 potential couples; (**b**) MHC-II, 83 real couples were compared with 258 potential couples. The transparency and the slight dispersion of data points around their values were used to improve readability.

We first used Generalized Linear Mixed Models (GLMMs) to investigate whether females chose sires of their offspring based on MHC dissimilarity within the couple or based on MHC diversity of the male. For this and all other female choice tests, we assumed each offspring to be the result of one “choice” event (more details in the Methods section). We used the following five MHC genetic parameters (described in detail under Methods): (i) male allele dissimilarity (MALDis); (ii) couple allele dissimilarity (CALDis); (iii) mean amino acid dissimilarity (μAADis); (iv) male allele diversity (MALDiv) and (v) male amino acid diversity (MAADiv). In other words, we asked if the probability of any given male to be a father correlated with any parameter above. We found that the probability of males to sire offspring increased significantly with increasing MALDis, both for MHC-I (p-value = 0.0099) and MHC-II (p-value = 0.0310). The odds of being chosen increased from 6% to 24.5% for MHC class I and from 22% to 53% for MHC-II, between the least and the most dissimilar couples (Fig. 1). No effect was detected for CALDis (p-values = 0.8810 and 0.2577 for MHC-I and MHC-II, respectively) or μAADis (p-values = 0.1098 and 0.2282 for MHC-I and MHC-II, respectively). We obtained strong evidence for a correlation between the probability for males to be chosen and their MHC diversity, both on the allele level (MALDiv, p-values = 0.0009 and 0.0059 for MHC-I and MHC-II respectively) and on the amino acid level (MAADiv, p-values = 0.0009 and 0.0021 for MHC-I and MHC-II respectively). For MHC-I, the probability of being chosen increased from 6.5% to 21% (MALDiv) and from 6% to 32% (MAADiv) between the least and the most diverse males in our sample (Fig. 1a). For MHC-II, the probability of being chosen increased from 23.5% to 51% (MALDiv) and from 23% to 55% (MAADiv) between the least and the most diverse males in our sample (Fig. 1b).

By replicating the allele-based analyses (MALDis, CALDis and MALDiv) using eight independent microsatellite markers, we found no evidence for a relationship between the probability of males being chosen by females and microsatellites (p-values = 0.33, 0.87 and 0.61 for MALDis, CALDis and MALDiv, respectively).

### Test of MHC-dependent female mate choice with randomizations

**Figure 2:**
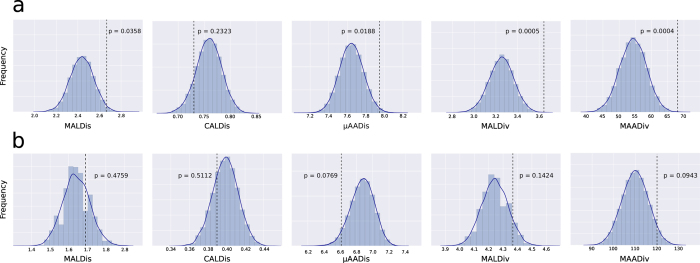
MHC-dependent female choice tested with Monte Carlo simulations. Frequency distributions of MHC index means calculated from 10^5^ Monte Carlo randomizations (blue bars) of potential male-female couples out of the sample studied, concerning the five MHC genetic indices. The mean values for the real couples (MALDis, CALDis and μAADis) or real fathers (MALDiv and MAADiv) are given in each plot by a dashed vertical line and the corresponding p-values are indicated. (**a**) MHC-I, 50 real couples were compared with 351 potential couples; (**b**) MHC-II, 83 real couples were compared with 258 potential couples.

We also ran Monte Carlo randomizations^43^ to test whether females chose males that were significantly more dissimilar or diverse to them than males randomly chosen from the pool of candidate mating partners. Concerning MHC class I (Fig. 2a), we found evidence for disassortative female choice testing MALDis and μAADis (p-values = 0.0358 and 0.0188, respectively) but not CALDis (p-value = 0.2323). We found evidence for female preference for more MHC-I diverse males by testing the two diversity parameters: MALDiv and MAADiv (p-values = 0.0005 and 0.0004, respectively). Concerning MHC class II (Fig. 2b), we could not reject the null hypothesis of randomness regarding the three dissimilarity indices MALDis, CALDis or μAADis (p-values = 0.4759, 0.5112 and 0.0769, respectively) or the two diversity indices MALDiv and MAADiv (p-values = 0.1424 and 0.0943, respectively).

### Role of TAAR genes in female choice

**Figure 3:**
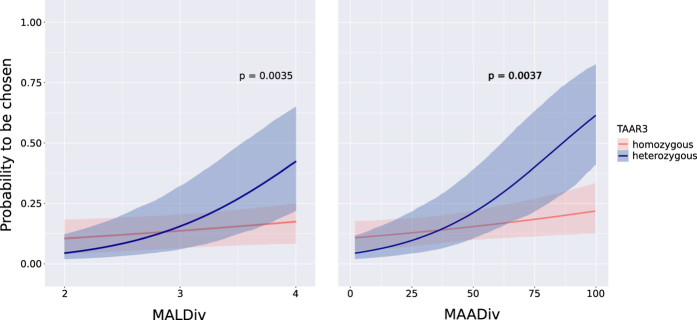
Effect of heterozygosity at the TAAR3 locus among females on their probability to choose males based on MALDiv and MAADiv indices at their MHC-I. The p-values corresponding to the interaction between heterozygosity status and MHC diversity index are given above the curves, which represent heterozygous (blue) and homozygous (orange) females at the TAAR3 locus. All females involved in couples genotyped for MHC-I were also genotyped for TAAR3.

We then investigated if the diversity at TAAR2, TAAR3 or TAAR8 among breeding females had an effect on the observed non-random MHC-dependent mating patterns described above. We found evidence for an interaction between the heterozygosity status of TAAR3 and both MALDiv and MAADiv (p-values = 0.0035 and 0.0037, respectively, Fig. 3). This led us to test the interaction between these two MHC diversity indices and females’ TAAR3 individual alleles. Here we found the presence of the allele TAAR3-2 and both MALDiv and MAADiv (p-values = 0.0485 and 0.0416, respectively) to be correlated.

In order to test if this interaction was a result of female choice or of a sampling artifact, we compared MALDiv and MAADiv of males that were candidate mating partners for TAAR3-homozygous females and of those that were candidate mating partners for TAAR3-hetorozygous females. Besides of sharing 89% of individuals, both groups were found to be nearly identical concerning MHC-I diversity (details in the online Supplemental Fig. S5). Finally, we tested if any *a priori* relationship between female TAAR3 heterozygosity and MHC-I diversity biased the interaction described above and found no evidence for it. Female MHC-I allele diversity or amino acid diversity showed no evidence for a correlation with female TAAR3 heterozygosity (p-values = 0.228 and 0.960, respectively).

A borderline interaction was found between TAAR2 constitution and MAADiv of MHC-I (p-value = 0.0549). No evidence of interaction was detected involving MHC class II or TAAR8 loci (p-values > 0.11).

## Discussion

In line with our expectations, our results indicate that female *S. bilineata* consider the MHC when they choose a mate; they prefer males with a high diversity of MHC class I alleles and males with MHC class I alleles that are dissimilar to their own. These results are supported by two different and independent statistical approaches (linear modelling and randomization tests). Linear modelling provided evidence that female S. bilineata also prefer males with a high diversity of MHC class II alleles and males with MHC class II alleles that are dissimilar to their own. Furthermore, female choice in this species was influenced by female’s genotypes at the TAAR3 locus. This is the first study reporting MHC-dependent mate choice and TAAR allele diversity among bats, and the first investigation showing a genetic background for the link between the MHC and a chemosensory receptor in any species.

By fitting models to our data, we found that the probability of female choice for a specific male depended on the parameters MALDis, MALDiv and MAADiv, both concerning class I and class II MHC genes (Fig. 1). The linear models enabled us to additionally calculate effect sizes for each explanatory parameter. We found that the odds to be chosen increased approximately two to four-fold both from the most similar to the most dissimilar male, as well as from the least to the most diverse male. In all cases, the relative increase in probability depending on MHC indices was stronger for MHC-I than for MHC-II.

By comparing our data to a set of 10^5^ Monte Carlo randomizations (Fig. 2), we observed strong evidence of non-random, disassortative mating concerning MHC class I genes (Fig. 2a). We could not rule out random mating for MHC class II genes (although sample size was higher). Disassortative female choice for MHC-I has been observed in many species before^5^. In our case, the particularly high prevalence of viruses in bats^38^ may explain the apparently prominent role of MHC-I over MHC-II concerning female choice.

We further found evidence that the TAAR3 locus can affect MHC class I-dependent female choice. Specifically, the relationship observed between the diversity parameters (MALDiv and MAADiv) and the probability of a male to be chosen by a female is significantly influenced by the heterozygosity or homozygosity at the TAAR3 locus in females. It seems therefore possible that the chemical signals involved in the recognition of MHC class I diversity of males are ligands of TAAR3 alleles. Thus, heterozygosity is likely to increase the sensory resolution of females for this kind of chemical communication. As depicted in Fig. 3, females that were heterozygous for TAAR3 were up to three times more likely to choose the most diverse male than homozygous females (for MALDiv and MAADiv, respectively, but compare also with Fig. 1a for females in general).

Given the fact that very little is currently known about TAAR ligands^26^ and that these are likely to be important for chemical communication^23^,^24^, we encourage subsequent work to consider the TAAR3 alleles identified here as candidates for binding odorants relevant for mate choice. The most likely alleles to play a role in our case are TAAR3-1 and TAAR3-2, as they were the common TAAR3 alleles (abundance of 89.5% and 54.8%, respectively). By testing the interaction of TAAR3-1 and TAAR3-2 alleles separately with the diversity parameters MALDiv and MAADiv, we found evidence that the females with TAAR3-2 were more likely to choose MHC-I diverse males as mates. On the other side, presence of TAAR3-2 and TAAR3 heterozygosity were strongly collinear (R^2^ = 0.91), which makes it difficult to disentangle the effect of TAAR3-2 alone. Although we have detected one single non-synonymous genetic polymorphism between TAAR3-2 and TAAR3-1, other substitutions are likely to be harbored outside of the area assessed in this work, since we have sequenced a relatively short segment of the coding region. Moreover, our LD results indicate physical linkage among the three TAAR loci investigated here. It is therefore likely that haplotypes spanning more than one locus (as for example further TAAR loci not investigated) segregate in this population. This seems to be the rule in other vertebrates^44^. We therefore speculate that, although we could not reject the null hypothesis of randomness for TAAR2 and TAAR8, polymorphisms in these and other neighboring loci can be part of haplotypes that influence females’ preference for MHC-I diverse males.

Concomitant with sexual maturation, females of *S. bilineata* immigrate to colonies in which they are unrelated to the resident males^36^. Females are thus, at first, potentially “equidistant” from all males in a colony, regarding kinship. Over time and generations, however, the probability increases for females to meet their sexually mature sons, grandsons and other “second degree” relatives. In these cases, they seem to avoid pairing with close relatives and the high frequency of extra-harem paternities has been explained before as a way to avoid inbreeding^45^. The combination of dispersal with inbreeding avoidance can theoretically lead to disassortative mating patterns similar to those observed for the MHC in this study. However, the disassortative pattern is not detectable regarding microsatellites. Microsatellites are particularly well suited as an internal control for genetic patterns driven by inbreeding avoidance due to neutrality. Although the lack of statistical significance for an association between microsatellites and female choice does not “prove” the absence of an association, it strengthens evidence, when compared to the MHC results, that the mating preferences that we observed are, indeed, targeted to the MHC and not to genome-wide dissimilarity or diversity.

As stated before, instead of assigning MHC alleles to specific loci, we considered them, in each class, as alleles from a single locus. This limitation is common in studies with non-model species^46^,^47^ due to lacking knowledge about their genomic structure. The methodological approach established in this work (see full protocol in the supplemental experimental procedures) offers a novel framework for other investigations that use large datasets involving non-model species in ecological contexts, in the absence of replicates. We adapted a clustering algorithm to perform allele calling and used two independent and complementary statistical approaches to assess MHC-dependent mate choice. We believe these bioinformatics and statistical tools to be valuable for other analyses, in particular on non-model species.

MHC-dependent mate choice has been tested with randomization tests relatively often elsewhere^14^,^19^,^43^. These tests investigate the probability that certain genetic parameters (such as MHC diversity) correlate with female choice more strongly than parameters observed under simulated panmixia. As such, they differ from (and complement) the modelling tests that estimate whether, and to which extent, MHC genetic parameters actually predict the probability of a male being chosen by a female. These tests, although based on the same dataset, are independent approaches that investigate similar consequences of female choice. By using the mating outcome (offspring) of naturally paired individuals as a proxy for mate choice, our analyses are “blind” to postcopulatory female choice phenomena such as selective abortion. There is however currently no evidence for cryptic or postcopulatory female choice in *S. bilineata*^35^,^37^,^45^,^48^. Likewise, male coercion has never been observed. The fact that we found TAAR3 associated with mating preferences reinforces the notion of odor-mediated (and therefore precopulatory) female choice in this species.

Finally, we have highlighted one specific TAAR locus with a high potential of being involved in mate-choice. Substantial progress has been recently made towards the deorphanization of certain groups of odorant receptors^49^ and knowledge about the peptide sequence of specific receptors likely involved in mate choice can accelerate this development. The large number of chemosensory receptor genes present in vertebrate genomes makes it evident that a well-established list of candidate receptors (and putative ligands) is pivotal for future studies investigating chemical communication involved in mate choice processes. From an evolutionary perspective, identifying the proximal mechanisms underlying mate choice is a necessary step towards understanding the evolution of mate choice and the diversity of mating strategies among animals.

## Methods

### Field and laboratory methods

#### Study species, fieldwork and paternity analyses

All bats (n = 981) were captured in the area of the ‘La Selva’ field station of the Organization for Tropical Studies (OTS) in Northeastern Costa Rica, as described elsewhere^45^. Collection of samples (wing punch) took place during sixteen years (1996 to 2011) in 11 day roosts. All bats were banded with color-coded plastic rings for identification. Females give birth to one pup per year and live up to 11 years^36^,^50^. Out of the 185 females investigated which sired offspring, 97 had between two and nine pups (average + – SD = 3.06 +− 1.31 pups/female). Females with more than one pup reproduced with an average of 2.3 males (SD = 0.8). Litters with more than one offspring were never observed^36^. Group structure (colony sizes, identities of individuals, motherhood of juveniles) was determined daily to weekly in the study day roosts during the parturition (June-August) and mating periods (November-January)^48^,^50^. Observation data from the mating season was available for eight years. For most offspring, the set of candidate mating partners was determined based on mating season observation data. It included all males older than 1.5 years observed or assumed to be roosting in the colony during the mating season in which each offspring was conceived (mean number per offspring + − SD = 9.6 + − 8.1, range = 2–21). When observational data was unavailable, the set of candidate mating partners was determined from previous and subsequent mating season combined with capture and paternity information. Paternity assignment had been conducted previously based on 11 microsatellite loci^51^ (three of which were excluded from the present analysis because they showed borderline evidence of linkage disequilibrium with other markers). Maternities were determined by the same microsatellites and by nursing observations^48^. Further details on DNA sampling and paternity assignment were published before^30^,^37^,^48^. Work permits were granted by the OTS. Research permits were granted by the MINAE (Ministerio del Ambiente y Energia) and the ACCVC (Área de Conservación Cordillera Volcánica Central). Animal treatment followed the Guide for Care and Use of Laboratory Animals of the National Institutes of Health. Animal handling complied with current Costa Rican laws and was approved by the Animal Care Review Committee of the SINAC (Sistema Nacional de Áreas de Conservación) and MINAE (permits 272-2003-OFAU, 135-2004-OFAU, 022-2005-OFAU, 108-2006-SINAC, 147-2007-SINAC, 183-2008-SINAC, 187-2009-SINAC, 130-2010-SINAC and 068-2011-SINAC).

#### Amplicon production and sequencing

We performed large-scale amplicon sequencing on the Illumina Genome Analyzer IIx platform to genotype variable regions of MHC class I and class II loci (exon 2), TAAR2, TAAR3 and TAAR8. Amplicon sizes, exon length, primer sequences and wet lab details are provided in the Supplemental Tables S1 and S2. For primer design, we combined sequences produced by cloning of ten unrelated individuals with public DNA sequence data^44^ from homologous regions from *Myotis lucifugus, Pteropus vampyrus* and, when necessary, other vertebrate species. For the MHC, we tested multiple primer pairs in or flanking the exon 2 and also analyzed blood transcriptome information of one individual. We aimed at the amplification of homologues of human HLA-A or HLA-B (MHC class I) and at the amplification of homologues of human HLA-DRB1 (MHC class II). For the TAAR genes, no public information was available for the two chiropterans mentioned. We therefore based primer design on alignments of TAAR sequences retrieved from dog, cow, mouse and human through the GenBank^44^. The combination of primer pairs for the loci TAAR2, TAAR3 and TAAR8 was the only one providing satisfactory amplification results while being multiplexed and diverse enough in terms of TAAR families. This determined our decision of which TAAR loci to genotype. Amplicons were purified, quantified and pooled (41 pools of 24 individuals each, three additional pools of repetitions and one pool of replicates) for sequencing. Barcoding (list given in the Supplemental Table S3) was employed for distinguishing individuals within each pool. Each individual sample was amplified separately and each amplicon pool contained one unique barcode combination.

#### Quality check and allele calling

Since replicates were included for only 24 samples due to limited DNA quantity, we developed a novel bioinformatics workflow instead of using our recently described approach^52^. After removing low-quality reads and reads with sequencing errors on primer or barcode sequences, reads were submitted to a chimera filter^53^ (default settings). Sequences passing it were submitted to a BLASTx search against known MHC or TAAR sequences^44^. Alignments of reads passing BLAST from all individuals, from each amplicon, were produced with Geneious7 Read Mapper. Allele calling was performed with Oligotyping^54^, which allows distinguishing very similar alleles based on Shannon diversity (SNP allele frequency, in our case). This made it possible to disentangle “real” alleles from a background of variation attributed to sequencing errors. Dedicated Python scripts were employed to assign alleles to individuals, while removing putative alleles present in one single individual and removing individuals that did not yield the minimum number of alleles. Replicates were handled blindly. The detailed bioinformatics pipeline is described in the supplemental material.

### Statistical methods

#### Linkage Disequilibrium

We measured LD between all genotyped loci using the exact test for LD implemented in Arlequin^55^ with 10^5^ Markov chain and 10^4^ dememorization steps. This test is an extension of Fisher’s exact test to contingency tables of arbitrary sizes and it allows detecting LD between pairs of loci. The test’s p-value is calculated as the proportion of generated tables (10^5^) with a smaller or equal probability to the observed contingency tables. The exact test of population differentiation was applied as implemented in Arlequin.

#### MHC genetic parameters

We measured three different parameters of MHC dissimilarity within each bat couple and two parameters of MHC diversity for each male.

1. Male Allele Dissimilarity (MALDis): number of MHC alleles present in the male which are not shared by the female. In other words, it measures, for each couple, the number of alleles “unknown” to the female.
2. Couple Allele Dissimilarity (CALDis): sum of the number of non-shared MHC alleles in each couple.
3. Mean Amino Acid Dissimilarity (μAADis): mean amino acid distance (measured as the number of amino acid differences) among the non-shared MHC alleles in each couple.
4. Male Allele Diversity (MALDiv): number of different MHC alleles present in each male.
5. Male Amino Acid Diversity (MAADiv): sum of amino acid distances among the MHC alleles of each male.

#### MHC-dependent female choice for mates with GLMMs

We used GLMMs to test if and how well an MHC genetic parameter could predict the probability of a male to be the father of the offspring of a female. We assumed each offspring as the outcome of a female choice event and tested if the probability of a male being chosen by a female was correlated to any of the five MHC indices described above. We calculated, for each offspring, all five MHC genetic parameters corresponding to its real parents (real female choice) and each of its potential parents (potential female choices). The calculated results of the MHC genetic parameters were organized in one row per female choice and the value of a binary variable called “CHOSEN” was set to “1” or “0”, depending on the couple being real or not, respectively. Thus, the final dataset consisted of 133 rows with a “1” in the column “CHOSEN” (number of sampled offspring to which a father was assigned and for whom at least one further candidate father was genotyped) and 476 lines with a “0” in the column “CHOSEN” (sum of the number of candidate fathers assigned to all offspring). We considered the colonies, parturition years, as well as identities of the males and females as four Gaussian random effects. The GLMMs were fitted using the R package spaMM^56^. In R syntax, the structure of the tested models was:

HLfit(cbind(CHOSEN,1-CHOSEN)~MHC_INDEX+ (1|COLONY) + (1|YEAR) + (1|CANDIDATEFATHER) + (1|MOTHER),family=binomial), where “MHC_INDEX” is one of the five MHC genetic parameters. Significance of “MHC_INDEX” was assessed by a Likelihood Ratio Test (LRT) comparing the likelihoods of a model with and a model without it. Specifically, p-values were computed after comparing the LRT statistic to its distribution under the null hypothesis. This distribution was generated by performing 1,000 parametric bootstrap replicates using the spaMM function *fixedLRT*, with the arguments *HLmethod* set to “ML” and *family* to “binomial()”. The 95% confidence intervals were generated by 1,000 bootstrap replicates.

#### Randomization tests

We investigated the relationship between the five MHC genetic parameters and mate choice by comparing the observed means of each index to randomized means obtained by Monte Carlo simulations^14^,^43^. We first calculated a mean of the MHC genetic indices for all real couples. We then created a distribution for each MHC genetic index by disassembling all couples and reassembling them randomly 10^5^ times (with replacement and using, for each offspring, its mother and the set of candidate males available to her, including the father). Two-tailed P-values were calculated directly as the proportion of times the simulated means were equally or more distant from the center of the distributions than the observed mean was.

#### Influence of TAAR genes on female choice for mates

We evaluated the influence of females’ TAAR2, TAAR3 and TAAR8 genes upon their choosing behavior for mates. Our hypothesis was that TAAR diversity influenced the ability that females have to select mates based on MHC dissimilarity or male diversity. Thus, we tested if there was an interaction between TAAR loci and the MHC parameters that had been found to be correlated with the probability of a male being chosen. To avoid multiple testing and overparameterized models, we considered each TAAR locus as a factor and its homozygosity or heterozygosity status as levels of that factor. We then tested the significance of the interaction of the factor “TAAR locus” with MALDis, MALDiv and MAADiv. In R syntax, the structure of the test was anova(M0, M1, boot.repl=1000) and the models were: M0 <- HLfit(cbind(CHOSEN, 1-CHOSEN) ~ MHC_INDEX*factor(TAARlocus) + (1|COLONY) + (1|YEAR) + (1|CANDIDATEFATHER) + (1|MOTHER), family="binomial", HLmethod = "ML", control.HLfit = list(max.iter=10000)) and M1 <- HLfit(cbind(CHOSEN, 1-CHOSEN) ~ MHC_INDEX+factor(TAARlocus) + (1|COLONY) + (1|YEAR) + (1|CANDIDATEFATHER) + (1|MOTHER), family="binomial", HLmethod = "ML", control.HLfit = list(max.iter=10000)), where “MHC_INDEX” was MALDis, MALDiv and MAADiv. In the case for which a significant interaction between a TAAR locus and an MHC parameter was found, we tested further the interaction of the MHC parameters with presence or absence of individual alleles (same model structure, alleles as levels of factor “TAARlocus”).

### Data Accessibility

Further data on methods and results (sequence alignments, bioinformatic pipeline and methodological details) are available through the online supplemental information file. Python (bioinformatics and plotting) and R (statistics and plotting) scripts will be made available to all interested researchers upon request. Requests should be addressed to PSCS (pablo.santos@uni-ulm.de) or to SS (simone.sommer@uni-ulm.de).

## Additional Information

**How to cite this article**: Santos, P. S. C. *et al*. MHC-dependent mate choice is linked to a trace-amine-associated receptor gene in a mammal. *Sci. Rep.* **6**, 38490; doi: 10.1038/srep38490 (2016).

**Publisher's note:** Springer Nature remains neutral with regard to jurisdictional claims in published maps and institutional affiliations.

## Supplementary Material

Supplementary Information
