# Supplementary material for: MHC-dependent mate choice is linked to a trace-amine-associated receptor gene in a mammal
Source: Sci Rep. 2016 Dec 12;6:38490. doi: 10.1038/srep38490 (PMC5150237; doi:10.1038/srep38490)
Supplement: Supplementary Information [file srep38490-s1.pdf]

MHC-dependent mate choice is linked to a trace-amine-associated receptor gene in a mammal

Pablo S. C. Santos, Alexandre Courtiol, Andrew J. Heidel, Oliver P. Höner, Ilja Heckmann,

Martina Nagy, Frieder Mayer, Matthias Platzer, Christian C. Voigt and Simone Sommer

Supplemental Experimental Procedures and Data

Table of Contents

**1.**	**DNA Extraction and Barcoded Amplicon Production**	**2**
1.1.	Table S1: Primer information	3
1.2.	Table S2: PCR conditions	4
1.3.	Table S3: List of barcodes used to assign PCR amplicons to individuals	5
**2.**	**Bioinformatics Pipeline for the Amplicon Sequencing Data Processing**	**5**
2.1.	Preparation of the raw files	5
2.2.	Quality filter	5
2.3.	Deduplication and singleton deletion	6
2.4.	BLAST filter	6
2.5.	Identification and removal of potential chimeric artifact sequences	7
2.6.	Mapping reads to a reference sequence	7
2.7.	Allele calling with entropy and Oligotyping analysis	7
2.8.	Mapping alleles to their original individuals	7
2.9.	Naming alleles	8
2.10.	Note on the uncertainty of next-generation sequencing data in a non-model species	8
**3.**	**Supplemental Results**	**9**
3.1.	Table S4: Names, sampling periods and sample sizes concerning the eleven day roosts that contributed individuals to this study.	**9**
3.2.	Figure S1. Geographic and genetic structure among the eleven day roosts that contributed individuals to this study.	**10**
3.3.	Table S5: Number of individuals genotyped for each allele found in *S. bilineata*	**11**
3.4.	Figure S2: Names, nucleotide and amino acid sequences of MHC-I alleles	**12**
3.5.	Figure S3: Names, nucleotide and amino acid sequences of MHC-II alleles	**13**
3.6.	Figure S4: Names, nucleotide and amino acid sequences of TAAR alleles	**14**
3.7.	Figure S5. Comparison between males that were available for TAAR3-homozygous and TAAR3-heterozygous females.	**15**
3.8.	Figure S6. Collinearity among the five MHC indices	**16**
**4.**	**Supplemental References**	**17**

1. DNA extraction and barcoded amplicon production

All wing punch samples were stored in ethanol. DNA extraction was performed using a commercial kit (First-DNA all tissue kit) by GEN-IAL GmbH (Troisdorf, Germany) according to the supplier's instructions. Amplicon production was carried out for each sample with four dedicated PCR reactions using the primers given in Table S1.

PCR conditions are summarized in Table S2. We used long within-cycle extension times in order to minimize chimera generation. All primer sequences were designed for this work.

In order to track individuals after pooling PCR products for sequencing, we used 24 different molecular barcodes. These consisted of 4-base sequences added to the 5' end on the primers. The list of barcodes used is given in Table S3.

1.1. Supplemental Table S1: Primer information

Loci targeted	Amplified region*	Amplicon size (bp)	Approx. length of exon (bp)#	Ratio amplicon/exon	Primer name	Template-specific sequence (5' – 3')
MHC Class I	exon 2	265	288	0.92	MHC_ I_F	GCTCCCACTCCTGAGGTAT
					MHC_ I_R	CGCTCTGGTTGTAGTAGC
MHC Class II	exon 2	237	270	0.88	MHC_II_F	ACACAGAGGGTGCGGCTCCT
					MHC_II_R	GGAGGACACACCCGTGCACAA
TAAR2§	TM2, EC1, TM3, IC2, TM4 and EC2	306	717	0.43	TAAR2_F	TGGGCAGGAACTGGAACAAGCG
					TAAR2_R	GGCAGTCACTGATTTCCTCCTGGG
TAAR3§	EL1, TM3, IL2, TM6, EL3 and TM7	478	1032	0.46	TAAR3_F	TGCAAGTGGAATTGAAATACCCAAGCC
					TAAR3_R	ACCGATTTTATGCTGTGTGTCACCCT
TAAR8§	IL1, TM2, EL1, IL2, TM4 and EL2	353	1038	0.34	TAAR8_F	TGGATAATTCCTCCAGCCCGTCGT
					TAAR8_R	TGGAAACCTCCTGGTGATGATTGC

All primers were designed to anneal to (and amplify) exonic regions.

*Since TAAR genes have one single exon, the corresponding protein domains of the sequenced DNA are given for them. EL: Extracellular loop; TM: transmembrane domain; IL: intracellular loop. All domains are predicted based on amino acid sequence alignments with human or mouse homologue proteins. The G protein-coupled receptor structures 2Y03 and 1F88 of the PDB (http://www.pdb.org) were used as references.

# Lengths are based on annotated sequences of homologue exons from horse or domestic cat[1].

§ These primers were multiplexed in a single PCR for each individual.

1.2. Supplemental Table S2: PCR conditions

Genes targeted	Denaturation time (s)	Denaturation temperature (°C)	Annealing time (s)	Annealing temperature (°C)
MHC class I and II	45	95	30	58
TAARs	30	94	90	61.5

All PCRs were carried out in 35 reaction cycles with an initial denaturation of 3 min at 98°C, extension time of 60 seconds at 72°C and a final extension of 5 min at 72°C.

1.3. Supplemental Table S3: List of barcodes used to assign PCR amplicons to individuals.

Barcode #	Sequence
1	AACT
2	AAGA
3	ACCC
4	ACTT
5	AGAA
6	ATAC
7	ATCG
8	CAGG
9	CCAG
10	CCCT
11	CGCC
12	CGTT
13	CTTA
14	GAAT
15	GCTA
16	GGCA
17	GTAA
18	GTTC
19	TAAG
20	TATA
21	TCCG
22	TGGA
23	TTCA
24	TTGC

2. Bioinformatics Pipeline for the Amplicon Sequencing Data Processing

Here we aim at giving a detailed description on the bioinformatic pipeline that we employed, from the raw-data stage until allele-calling. This workflow is written in chronological order and in a "cookbook" form in order to facilitate comprehensibility and reproducibility. The comments, metrics and other results which are specific to the sequences produced in this work are shown separately and *in italics*. Except for when stated differently, all computations were performed in a dedicated Linux environment (http://environmentalomics.org/bio-linux). High performance computations were carried out in a system with 64 processing units and 512 GB of RAM space. DNA amplicon sequencing was performed in the Illumina Genome Analyzer IIx platform.

1st. Preparation of the raw files:

a. Compress (gzip) all FASTQ files (raw data) of all pools.
b. Produce FastQC (bioinformatics.babraham.ac.uk/projects/fastqc) reports and perform visual inspection in order to set exclusion parameters for the next step.

We processed data originated from 45 pools: 41 regular sample pools, 3 repetition pools and one pool of replicates. Each pool contained amplicons of 24 individuals which had been previously (during PCR) marked with one of 24 different barcode combinations (Table S3).

2nd. Quality filter:

a. Perform first quality filter using a Python script (all scripts available upon request) with FASTQ parameters "q30" and "p75", meaning that reads with FASTQ quality score < 30 in more than 25% of the bases in both directions are completely deleted.
b. Delete reads with clear artifacts (passages with repeats of A, C, G, T, GC or GT at least 15 bases long).
c. Delete reads with primer or tag errors (no error tolerance).
d. Delete obvious chimeras (reads with unexpected barcode combinations).
e. Check necessity of trimming out poor quality read ends.
f. Save sequences as FASTQ files and generate FastQC reports again for documentation.

We trimmed out 17 bases of the ends of all reads due to quality drop, meaning a Phred score below Q30 in over 50% of reads. At this point we selected the 80 individuals with the qualitatively poorest sequences. The DNA of these samples was amplified again (new amplicon production for all primer combinations) and re-sequenced in 4 repetition pools. We obtained 326,951,407 reads from sequencing, 146,532,367 (44.8%) of which were rejected due to low quality. Additional 92,169,875 reads (28.2% of total or 51.1% of the

remaining reads) were rejected because of primer/tag mismatches, leaving us with 88,249,165 (27.0% of total) reads.

3rd. Deduplication and singleton deletion:

a. Compare all sequences in a pool in order to identify duplicated (or redundant) sequences. This aims at decreasing the sizes of the files and is done with the Tally algorithm[2].

b. Delete all sequences with frequency of 1 (singletons).

c. Produce FASTA files with identifiers such as ">S12:C45". This identifies a specific sequence as "S12", present with 45 copies.

4th. BLAST filter:

a. Set up two local BLAST (blast.ncbi.nlm.nih.gov) protein databases with all publicly available vertebrate sequence information: (1) for MHC-I and II and (2) for TAARs. Fetch annotated sequences from the GenBank[1].

b. Parallelize local BLAST searches (BLASTx) over 64 computer processing units in order to compare every FASTA entry (step 3c) against the relevant database.

c. Parse BLAST results in order to generate "Hits" and "No-Hits" lists (set Expect Value threshold to 10^{-13}). The "no-hits" query sequences are now considered sequencing errors, artifacts, products of contamination or reads from other genomic regions.

d. Parse the list of positive BLAST results, and delete those with a premature stop codon or frame-disrupting substitutions (protein with less than 90% of expected length).

In addition to MHC and TAAR genes we also sequenced genome wide odorant receptor (OR) genes using highly degenerate primers. As read quality, read diversity and assembly method (see below) differed strongly for ORs, we decided not to keep them in the analysis. After excluding OR gene reads (ca. 50% of reads) and performing BLAST, a total of 14,173,773 reads were kept for further processing. The number of reads kept per gene was:

- *1,846,099 for MHC-I*
- *1,731,254 for MHC-II*
- *7,196,725 for TAAR2*
- *2,300,900 for TAAR3 and*
- *1,098,795 for TAAR8.*

5th. Identification and removal of potential chimeric artifact sequences:

a. Prepare files for UCHIME[3] using a dedicated Python script.

b. Run UCHIME[3], record output and remove sequences identified as possible chimeras from all FASTQ files.

6th. Mapping reads to a reference sequence:

a. Choose one reference sequence per gene.

b. Use Geneious7 Read Mapper[4] to produce large alignments of all information of all individuals for each amplicon. Trim out areas of consistently low (Q<30) quality. Save alignments as FASTA files.

7th. Allele calling with Entropy and Oligotyping[5] analysis:

Based on visual inspection, determine which positions in the alignment will be components of Oligotyping. Typically, a certain level of background entropy corresponding to sequencing errors is common to all positions in the alignment of an amplicon. The positions with higher-than-background entropy are considered as Oligotyping components as they are likely real polymorphisms.

8th. Mapping alleles to their original individuals:

Use a dedicated Python script which first parses the "matrix-count.txt" results of Oligotyping. Second, it assigns positive oligotypes to each individual. It then calculates frequency and abundance of each oligotype and excludes those with abundance of less than 2 individuals. It then counts the number of remaining oligotypes per individual and excludes individuals with less than 3 (MHC-I) or 2 (MHC-II) alleles. It repeats the last 2 steps until no allele or individual is removed.

We assigned:

- *50 MHC-I alleles to 447 individuals (3 to 6 alleles per ID)*
- *25 MHC-II alleles to 615 individuals (2 to 4 alleles per ID)*
- *9 TAAR2 alleles to 964 individuals (1 or 2 alleles per ID)*
- *5 TAAR3 alleles to 876 individuals (1 or 2 alleles per ID) and*
- *8 TAAR8 alleles to 884 individuals (1 or 2 alleles per ID).*

The number of individuals genotyped for more than one locus (information used later for linkage disequilibrium analyses) was as follows: 331 individuals were genotyped for both MHC classes, 829 individuals were genotyped for all 3 TAAR loci and 301 individuals were genotyped for all 5 loci studied here. 14 out of 24 replicate samples yielded enough reads for them to be used as such for all loci. Repeatability of allele calling was 88.2% on average (MHC-I: 75%; MHC-II: 81%; TAAR2: 95%; TAAR3: 90% and TAAR8: 100%).

9th. Naming alleles:

This final step depends on the nomenclature and conventions of the assessed species and genes. It generally should consider phylogenetic relationships between alleles and previously published allele names.

We named MHC class I alleles sequentially, according to the human nomenclature convention and using the "Sabi" prefix (for Saccopteryx bilineata).
*The same applies to MHC class II alleles, starting from Sabi-DRB*21. The alleles Sabi-DRB*1 to Sabi-DRB*20 have been described before*[6] *and are available through the Genbank under the accession numbers JQ388810.1 to JQ388829.1. Among the previously described Sabi-DRB alleles, we have found Sabi-DRB*02, Sabi-DRB*04, Sabi-DRB*07 and Sabi-DRB*20 in our sample.*
As no TAAR alleles were previously known for this species, we named alleles sequentially (for example TAAR2-1 to TAAR2-5), according to their frequency in this study.

10th. Note on the uncertainty of next-generation sequencing data in a non-model species:

On the one hand, the fact that we genotyped a non-model species which lacks a previously published reference genome makes it impossible to infer undoubtedly the number of MHC loci present. It is similarly not possible to be sure about structural variations such as copy number polymorphisms. On the other hand, these uncertainties do not affect the results of LD and mate choice analysis, provided that authors (i) find consistent results using independent measurements, (ii) find consistent results using different statistical approaches, (iii) use sample sizes that are large enough, (iv) make sure all error sources are evenly distributed over the sample and across both sexes, (v) are consistently conservative. The bioinformatics pipeline described here, as well as the statistical approach employed were designed to meet these criteria.

3. Supplemental Results

3.1. Supplemental Table S4. Names, sampling periods and sample sizes concerning the eleven day roosts that contributed individuals to this study.

Colony (day roost)	Sampling Years[1]	Nr of pups[1]	Nr of pups[2]	Nr of mothers[2]	Range of total number of males[2]	Total number of potential mate choices[2]
**BH**	1996-2011	197	173	67*	2-21	2,958 (88.3%)
**PH**	2003-2011	67	43	24	3-5	143 (4.3%)
**RH**	2004-2011	36	26	14	2-4	83 (2.5%)
**STR3000**	2007-2009	19	19	11	3-4	63 (1.9%)
**CG**	1998, 2001, 2004-2007, 2010-2011	31	17	17*	2-5	60 (1.8%)
**C2**	2003-2006	15	7	6	2	14 (0.4%)
**ZOM**	2007-2011	15	5	5	2-3	17 (0.5%)
**C5**	2004-2006	6	4	3	2-3	11 (0.3%)
**VB**	2010-2011	5	1	1	2	2 (0.1%)
**RS**	2004-2005	8	0	0	0	0
**STR1550**	2004	2	0	0	0	0
**Total**	**16 years**	**401**	**295**	**147***	**2-21**	**3,351**

1, 2: The numbers refer to the methodological filters employed (mother, father and at least one further candidate male had to be known and successfully genotyped) and indicate the individuals originally sampled (1) and those actually assessed for the mate choice analyses (2).

*One female that originally roosted in the CG colony immigrated into the BH colony, thus the number of females per colony adds up to 148 while the real total number of females is 147.

3.2. Supplemental Figure S1. Geographic and genetic structure among the eleven day roosts that contributed individuals to this study.

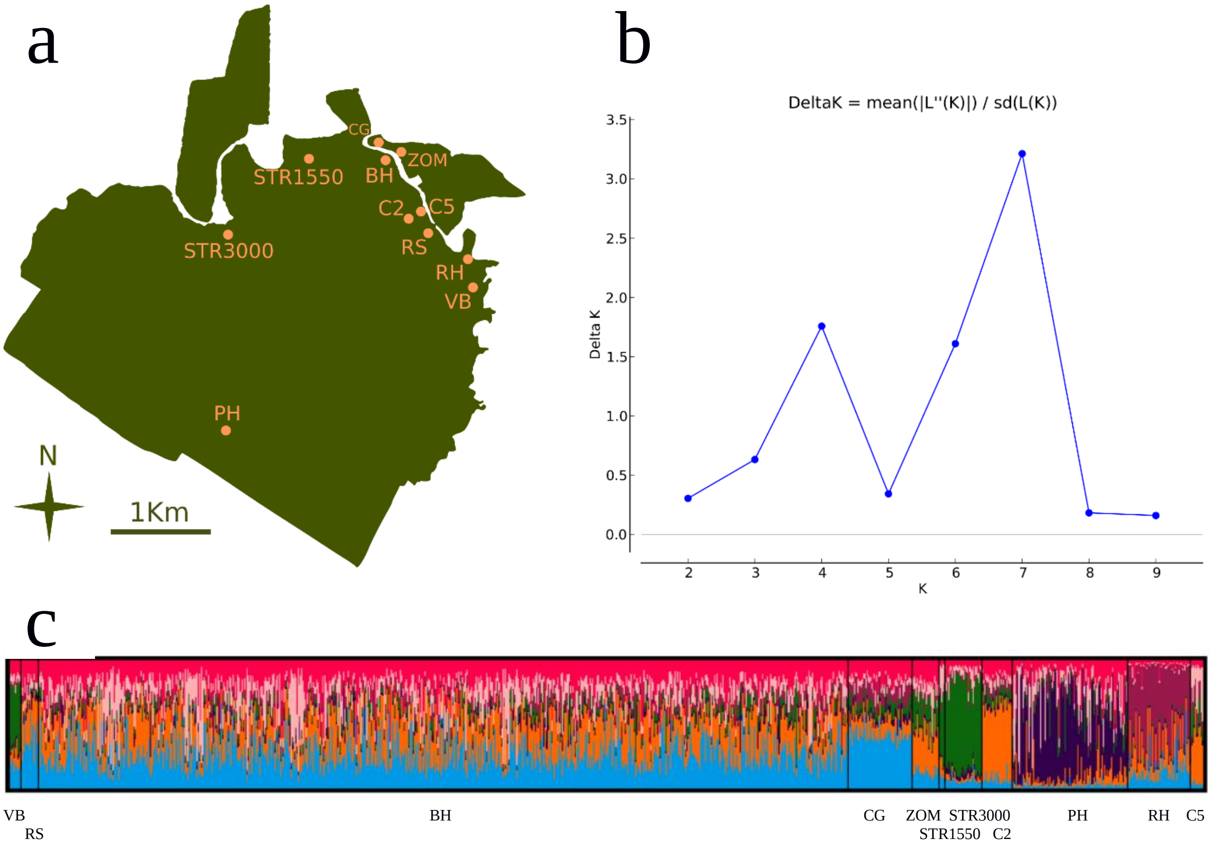

a: Simplified map of the *La Selva Biological Station* area in Costa Rica. The map was drawn with Inkscape[7] based on a reference map explicitly released into the public domain[8] in 2012.
b: ΔK plot comparing different numbers of assumed populations. The K value inferred to best fit the data was 7 (K = 1 up to K = 10 tested). The analysis was carried out with STRUCTURE (v2.3.4)[9] and the plot (Evanno method for detecting the number of K) was created with STRUCTURE Harvester[10].
c: STRUCTURE[9] bar plot in which the ancestry of individuals was inferred after averaging ten STRUCTURE[9] runs with CLUMPAK (*Cluster Markov Packager Across K*)[11] for K = 7. Each column represents one individual while each block includes all individuals from each colony, which are indicated below each block. Further parameters used were as follows: Burn-in period: 2.5 x 10^5; Replicates: 10^5; INFERALPHA=1; INFERLAMBDA=0.

The STRUCTURE analysis was performed based on eight microsatellites previously genotyped[12] for 1026 individuals, which include all bats genotyped for the MHC (see Table S4). It provides an idea about the relatively high degree of admixture, likely due to dispersion and gene flow, among the eleven colonies. As expected from their geographic locations, the colonies PH and STR3000 stand out by showing some degree of isolation, while BH seems highly influenced by its neighbors. Importantly, the differences among colonies do not affect mate choice tests, since females, real and candidate fathers are always from the same colony. Additionally, 88.3% of all mate choices investigated in our study took place in BH, the largest colony (Table S4).

3.3. Supplemental Table S5: Number of individuals genotyped for each allele found in *S. bilineata*

MHC-I (N = 447). 50 alleles	
**Symbol**	**Abundance**
Sabi-B*01	293
Sabi-B*02	142
Sabi-B*03	100
Sabi-B*04	72
Sabi-B*05	71
Sabi-B*06	66
Sabi-B*07	66
Sabi-B*08	62
Sabi-B*09	49
Sabi-B*10	48
Sabi-B*11	46
Sabi-B*12	45
Sabi-B*13	38
Sabi-B*14	35
Sabi-B*15	31
Sabi-B*16	27
Sabi-B*17	20
Sabi-B*18	20
Sabi-B*19	18
Sabi-B*20	17
Sabi-B*21	14
Sabi-B*22	13
Sabi-B*23	13
Sabi-B*24	12
Sabi-B*25	11
Sabi-B*26	11
Sabi-B*27	10
Sabi-B*28	11
Sabi-B*29	9
Sabi-B*30	8
Sabi-B*31	8
Sabi-B*32	8
Sabi-B*33	8
Sabi-B*34	7
Sabi-B*35	7
Sabi-B*36	7
Sabi-B*37	6
Sabi-B*38	6
Sabi-B*39	6
Sabi-B*40	5
Sabi-B*41	5
Sabi-B*42	5
Sabi-B*43	5
Sabi-B*44	5
Sabi-B*45	5
Sabi-B*46	4
Sabi-B*47	4
Sabi-B*48	4
Sabi-B*49	4
Sabi-B*50	3

MHC-II (N = 615). 25 alleles	
**Symbol**	**Abundance**
Sabi-DRB*02	28
Sabi-DRB*04	9
Sabi-DRB*07	482
Sabi-DRB*20	610
Sabi-DRB*21	448
Sabi-DRB*22	287
Sabi-DRB*23	187
Sabi-DRB*24	172
Sabi-DRB*25	99
Sabi-DRB*26	60
Sabi-DRB*27	59
Sabi-DRB*28	59
Sabi-DRB*29	30
Sabi-DRB*30	28
Sabi-DRB*31	21
Sabi-DRB*32	17
Sabi-DRB*33	15
Sabi-DRB*34	15
Sabi-DRB*35	15
Sabi-DRB*36	11
Sabi-DRB*37	9
Sabi-DRB*38	8
Sabi-DRB*39	8
Sabi-DRB*40	7
Sabi-DRB*41	4

TAAR2 (N = 964). 9 Alleles	
**Symbol**	**Abundance**
TAAR2-1	928
TAAR2-2	271
TAAR2-3	240
TAAR2-4	37
TAAR2-5	20
TAAR2-6	4
TAAR2-7	3
TAAR2-8	3
TAAR2-9	2

TAAR3 (N = 876). 5 Alleles	
**Symbol**	**Abundance**
TAAR3-1	784
TAAR3-2	480
TAAR3-3	16
TAAR3-4	7
TAAR3-5	6

TAAR8 (N = 884). 8 Alleles	
**Symbol**	**Abundance**
TAAR8-1	855
TAAR8-2	277
TAAR8-3	97
TAAR8-4	19
TAAR8-5	16
TAAR8-6	7
TAAR8-7	6
TAAR8-8	5

3.4. Supplemental Figure S2. Names, nucleotide and amino acid sequences of MHC-I alleles

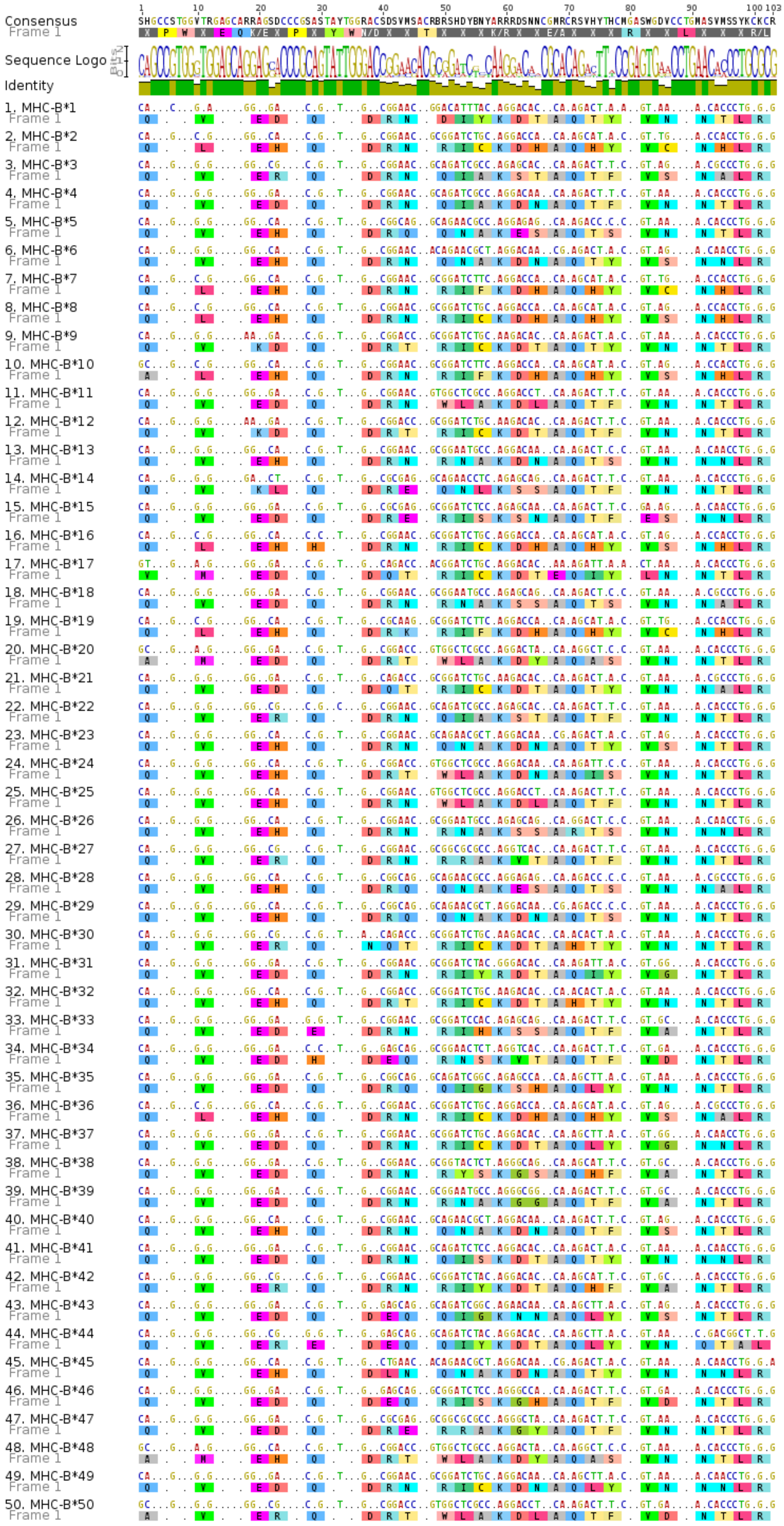

3.5. Supplemental Figure S3. Names, nucleotide and amino acid sequences of MHC-II alleles

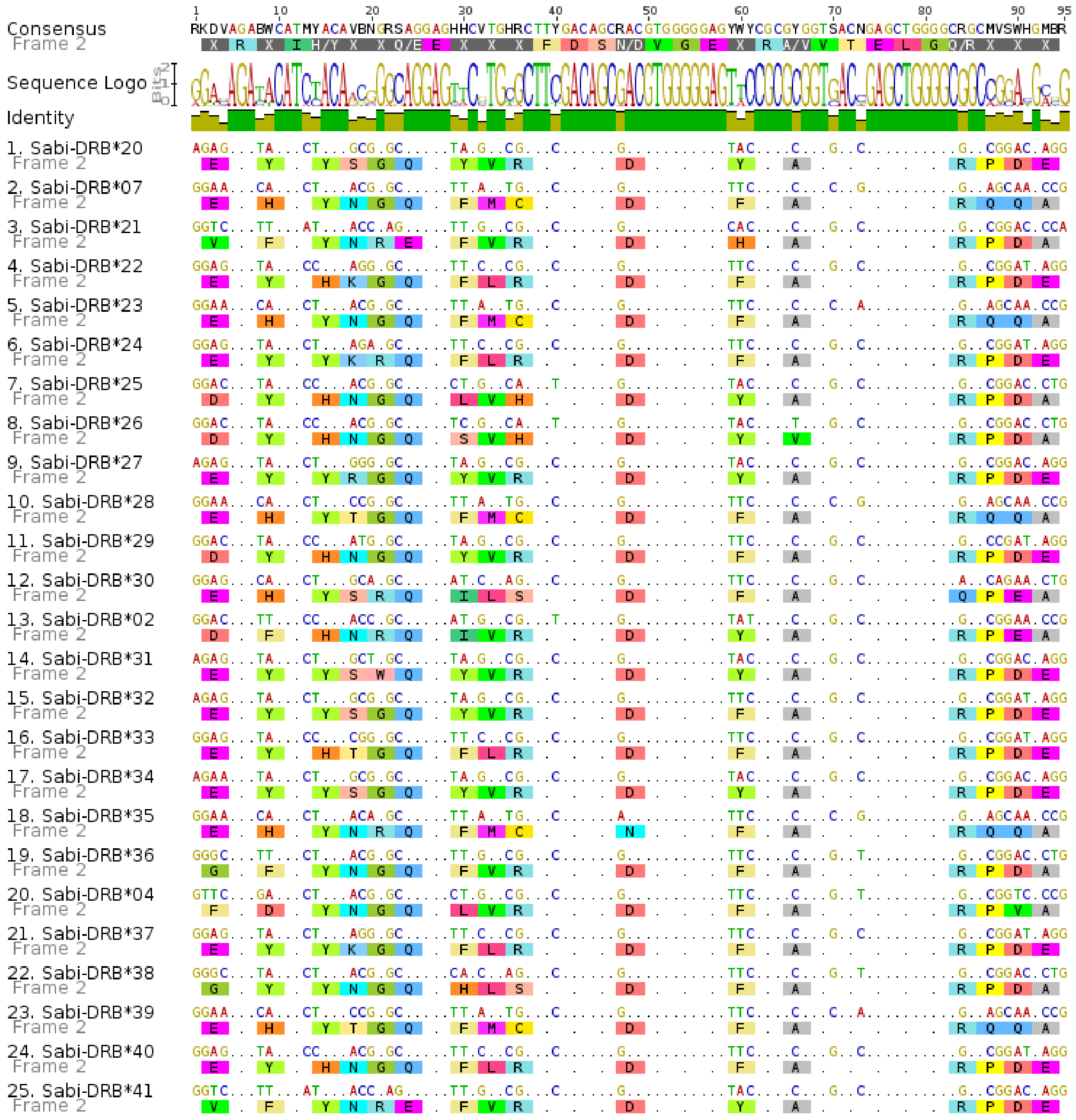

3.6. Supplemental Figure S4. Names, nucleotide and amino acid sequences of TAAR alleles

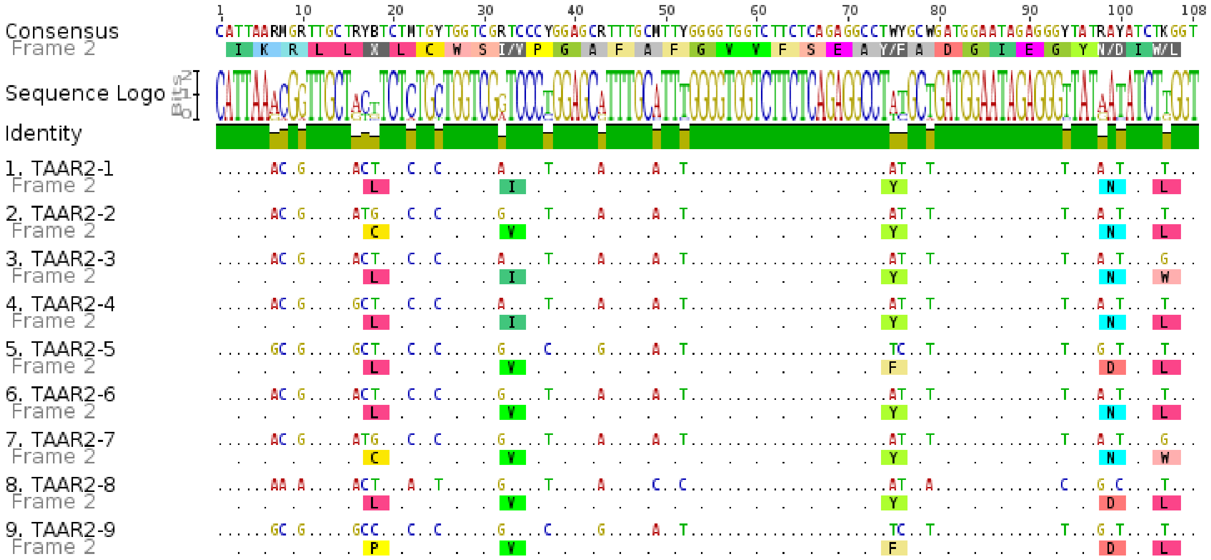

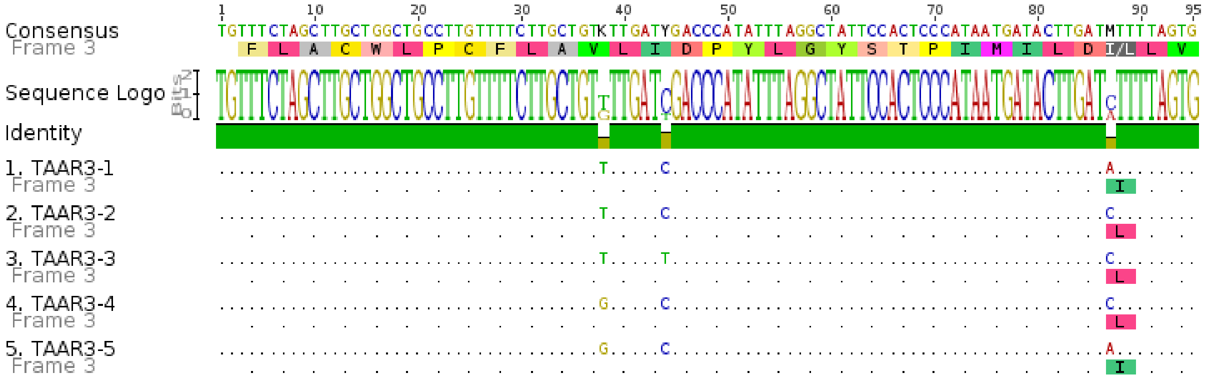

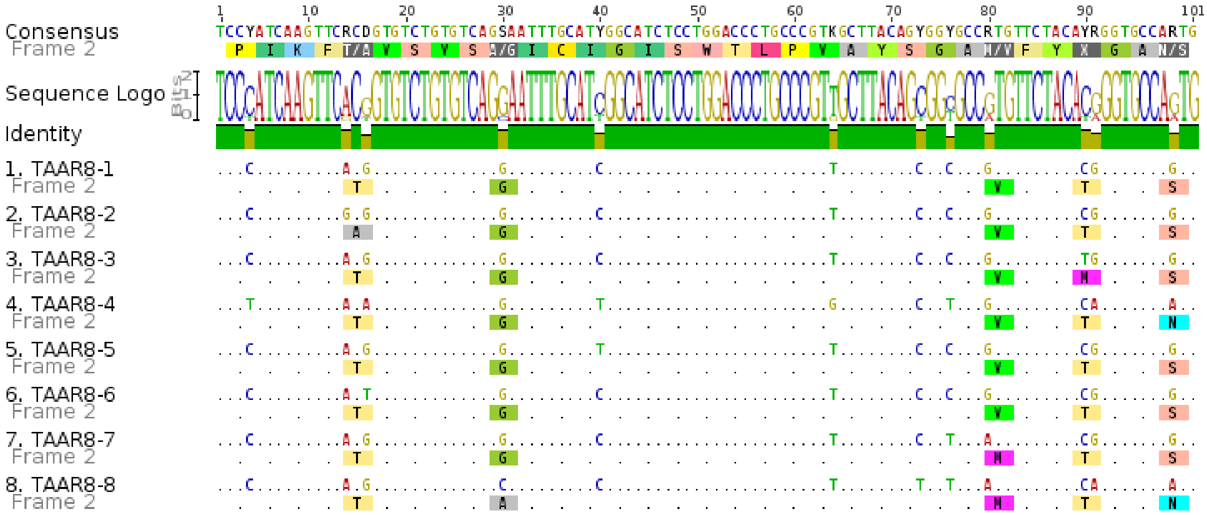

3.7. Supplemental Figure S5. Comparison between males that were available for TAAR3-homozygous and TAAR3-heterozygous females

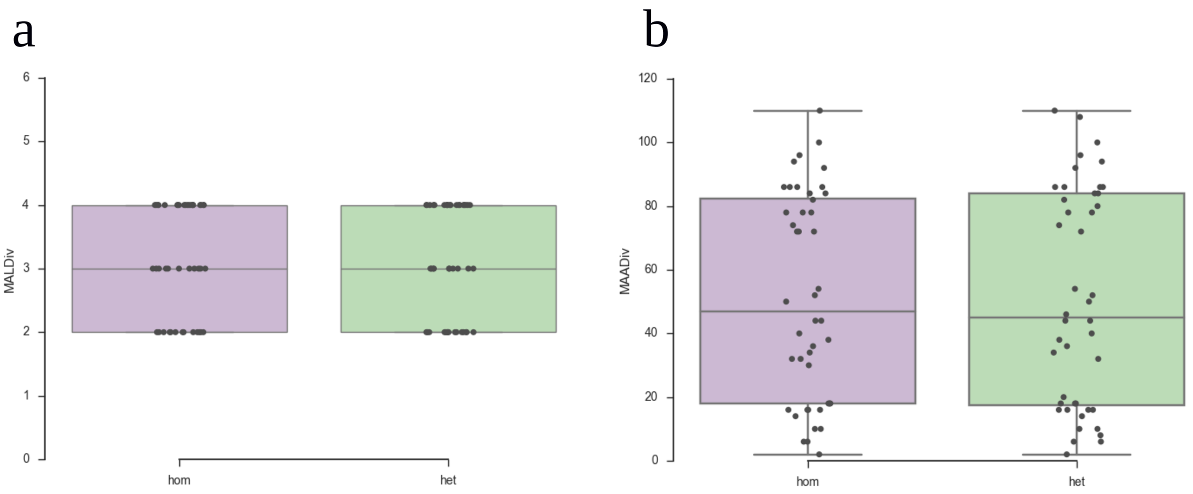

a: MALDiv analysis; b: MAADiv analysis; hom: 44 males that were candidate mating partners for TAAR3-homozygous females. het: 44 males that were candidate mating partners for TAAR3-heterozygous females. Five males in each group are exclusive and 39 are shared. There were 165 mothers genotyped for TAAR3 (87 homozygous and 78 heterozygous). The averages are as follows:
Average MALDiv of males available for homozygous females: 3.1591
Average MALDiv of males available for heterozygous females: 3.0910
Average MAADiv of males available for homozygous females: 51.4091
Average MAADiv of males available for heterozygous females: 50.9091

The distributions make evident that the two male groups have a nearly identical diversity indices. The slightly higher values among the “hom” group (males available for homozygous females) additionally corroborate the results of the TAAR3/female choice interaction analysis, which, if biased by male sampling, would be expected to be skewed in the opposite direction (higher MALDiv and MAADiv for mating partners of TAAR3-homozygous females).

3.8. Supplemental Figure S6. Collinearity among the five MHC indices

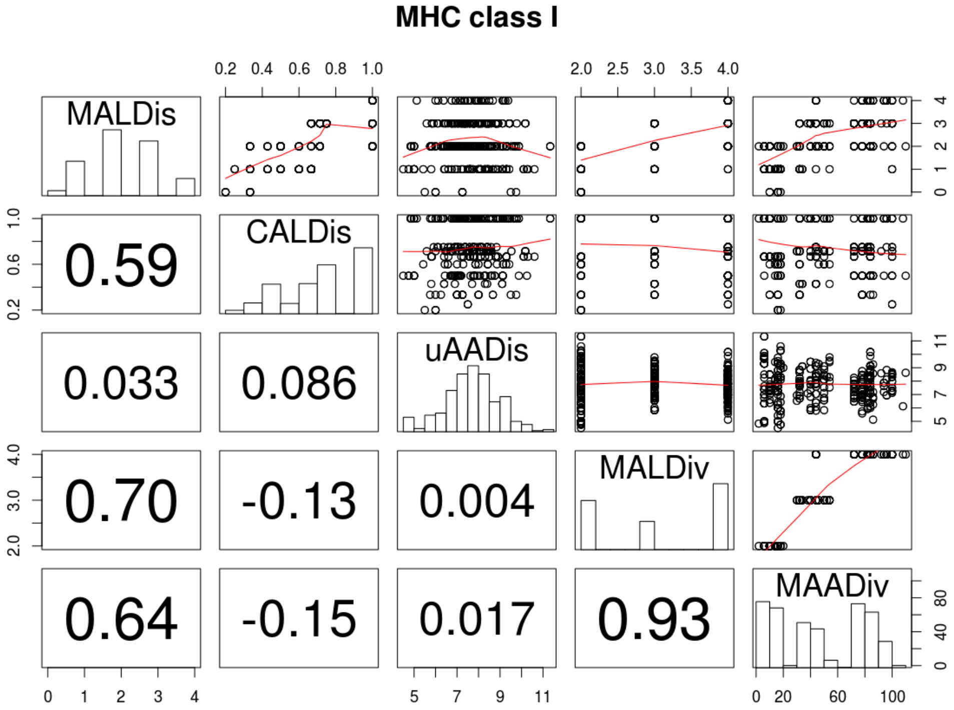

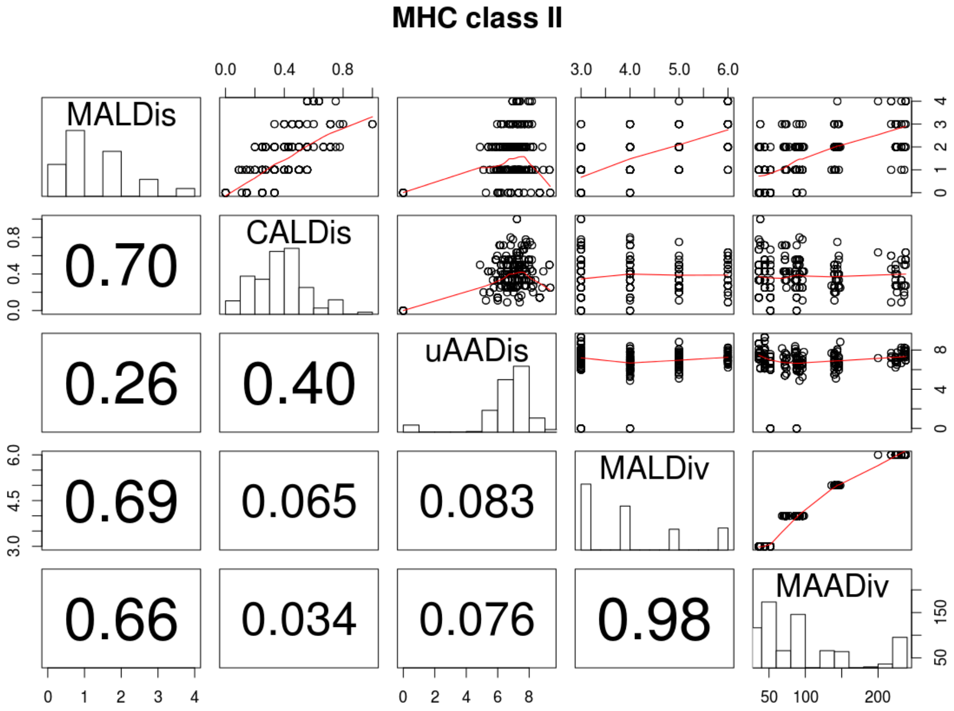

The lower panels display Pearson correlations (R^2) while the upper panels present scatter plots among each pair of variables (MHC indices) which are given in the diagonal.
For both MHC classes, collinearity is high among the diversity indices (MALDiv and MAADiv) but not among the dissimilarity indices (MALDis, CALDis and µAADis). The function for the plots was adapted from the *panel.cor* function published elsewhere[13].

4. Supplemental References:

1. Benson, D. A. *et al.* GenBank. *Nucleic Acids Res.* **41,** D36-42 (2013).
2. Davis, M. P. A., van Dongen, S., Abreu-Goodger, C., Bartonicek, N. & Enright, A. J. Kraken: A set of tools for quality control and analysis of high-throughput sequence data. *Methods San Diego Calif* **63,** 41–49 (2013).
3. Edgar, R. C., Haas, B. J., Clemente, J. C., Quince, C. & Knight, R. UCHIME improves sensitivity and speed of chimera detection. *Bioinformatics* **27,** 2194–2200 (2011).
4. Kearse, M. *et al.* Geneious Basic: an integrated and extendable desktop software platform for the organization and analysis of sequence data. *Bioinforma. Oxf. Engl.* **28,** 1647–1649 (2012).
5. Eren, A. M. *et al.* Oligotyping: differentiating between closely related microbial taxa using 16S rRNA gene data. *Methods Ecol. Evol.* **4,** 1111–1119 (2013).
6. Mayer, F. & Brunner, A. Non-neutral evolution of the major histocompatibility complex class II gene DRB1 in the sac-winged bat *Saccopteryx bilineata*. *Heredity* **99,** 257–264 (2007).
7. Inkscape. *SourceForge* Available at: https://sourceforge.net/projects/inkscape/. (Accessed: 15th September 2016).
8. WikiCommons. *GIS Map showing geology of La Selva Biological Station, Costa Rica. https://commons.wikimedia.org/wiki/File:Geology_in_La_Selva_Biological_Station,_Costa_Rica.jpg*. (2012).
9. Pritchard, J. K., Stephens, M. & Donnelly, P. Inference of Population Structure Using Multilocus Genotype Data. *Genetics* **155,** 945–959 (2000).
10. Earl, D. A. & vonHoldt, B. M. STRUCTURE HARVESTER: a website and program for visualizing STRUCTURE output and implementing the Evanno method. *Conserv. Genet. Resour.* **4,** 359–361 (2011).
11. Kopelman, N. M., Mayzel, J., Jakobsson, M., Rosenberg, N. A. & Mayrose, I. Clumpak: a program for identifying clustering modes and packaging population structure inferences across K. *Mol. Ecol. Resour.* **15,** 1179–1191 (2015).

12. Nagy, M., Knörnschild, M., Voigt, C. C. & Mayer, F. Male greater sac-winged bats gain direct fitness benefits when roosting in multimale colonies. *Behav. Ecol.* ars003 (2012).

13. Zuur, A. F., Ieno, E. N. & Elphick, C. S. A protocol for data exploration to avoid common statistical problems. *Methods Ecol. Evol.* **1,** 3–14 (2010).
